# Assessment of liver fibrosis in Egyptian chronic hepatitis B patients: A comparative study including 5 noninvasive indexes

**DOI:** 10.1097/MD.0000000000009781

**Published:** 2018-02-09

**Authors:** Mohammed Tag-Adeen, Maha Zeinelabedin Omar, Fatma Mohamed Abd-Elsalam, Ali Hasaneen, Mohamed Ahmed Mohamed, Hala Mohamed Elfeky, Ebada Mohamed Said, Badawy Abdul-Aziz, Amira Hefney Osman, Enas Sebaey Ahmed, Ghada Sidky Osman, Taghreed Abdul-Samie

**Affiliations:** a Department of Internal Medicine, Qena School of Medicine, South Valley University, Qena; b Department of Hepatology, Gastroenterology and Infectious Diseases; c Department of Internal Medicine, Faculty of Medicine, Benha University, Benha; d Department of Hepatology and Gastroenterology, Center of Cardiac and Digestive System Diseases, Sohag; e Department of Clinical Pathology, Faculty of Medicine, Benha University, Benha; f Department of Pathology, Qena School of Medicine, South Valley University, Qena; g Department of Pathology, Faculty of Medicine, Benha University, Benha, Egypt.

**Keywords:** AAR, APRI, chronic hepatitis B, Fib-4, noninvasive indexes, RPR, S-index

## Abstract

Fibrosis assessment in chronic hepatitis B (CHB) is essential for prediction of long-term prognosis and proper treatment decision. This study was conducted to assess predictability of 5 simple noninvasive fibrosis indexes in comparison to liver biopsy in CHB patients.

A total of 200 CHB adult Egyptian patients were consecutively included in this study, all were subjected to liver biopsy with staging of fibrosis using METAVIR scoring system. Fibrosis indexes including S-index, red cell distribution width to platelets ratio index (RPR), fibrosis-4 index (Fib-4), AST to platelets ratio index (APRI), and AST/ALT ratio index (AAR) were compared to biopsy result and their predictabilities for the different fibrosis stages were assessed using area under receiver operating characteristic curve (AUROC) analysis.

S-index showed the highest AUROCs for predicting fibrosis among the studied indexes. AUROCs of S-index, RPR, Fib-4, APRI, and AAR were: 0.81, 0.67, 0.70, 0.68, and 0.60 for prediction of significant fibrosis (F2–F4), 0.90, 0.66, 0.68, 0.67, and 0.57 for advanced fibrosis (F3–F4), and 0.96, 0.62, 0.61, 0.57, and 0.53 for cirrhosis (F4), respectively. The optimal S-index cutoff for ruling in significant fibrosis was ≥0.3 with 94% specificity, 87% PPV, and 68% accuracy, while that for ruling out significant fibrosis was <0.1 with 96% sensitivity, 91% NPV, and 67% accuracy. Accuracy of S-index was higher for predicting cirrhosis (91%) than that for predicting advanced fibrosis (79%) and significant fibrosis (68%).

S-index has the highest predictability for all fibrosis stages among the studied fibrosis indexes in HBeAg-negative CHB patients, with higher accuracy in cirrhosis than in the earlier fibrosis stages.

## 1 Introduction

Globally, 240 million persons have chronic hepatitis B infection (CHB) with the highest prevalence in Africa and Asia.^[1]^ It is estimated that the annual CHB-related deaths are 340,000 and 310,100 because of liver cancer and liver decompensation respectively.^[2–4,]^

An accurate assessment of liver fibrosis in patients with CHB infection is essential not only in determining whether and when to initiate antiviral therapy but also in predicting long-term clinical prognosis.^[4–6]^ Liver biopsy remains the gold standard for assessing liver fibrosis despite it has some limitations like invasiveness, sampling error, and variability in pathological interpretation.^[7–9]^ Furthermore, the dynamic process of liver fibrosis with the subsequent disease progression and regression cannot be easily quantified.

These limitations of liver biopsy have led to growing interest in the use of noninvasive methods including serum markers and transient elastography (TE) to assess hepatic fibrosis,^[6]^ and most attention has been focused on whether these methods can detect the presence or absence of significant fibrosis (F2), severe fibrosis (F3), and cirrhosis (F4) according to the METAVIR histological score.^[10–13]^ The aim of this study was to compare diagnostic validity of S-index, red cell distribution width to platelets ratio index (RPR), fibrosis-4 index (Fib-4), AST to platelets ratio index (APRI), and AST/ALT ratio index (AAR) as noninvasive fibrosis indexes in Egyptian HBeAg-negative CHB patients.

## 2 Ethical clearance

The study protocol adheres to the terms of the latest version of the Declaration of Helsinki for Medical Research and it was approved by the ethical committees of Benha Faculty of Medicine, Benha University, Egypt and the Egyptian Ministry of Health. A written informed consent was obtained from each patient before enrollment in this study.

## 3 Patients and method

### 3.1 Patients recruitment

This study was conducted in Benha University Hospital and Qena Fever Hospital, Egypt, between January 2016 and June 2017. All patients were already starting regular followed up visits in HBV out-patient clinic in both hospitals and underwent laboratory investigations and diagnostic liver biopsy as a routine procedure for identification of their fibrosis stage before starting or declining antiviral treatment. Patients have been selected based on laboratory and biochemical investigations which were then compared to biopsy result.

### 3.2 Inclusion criteria

Adult naive HBeAg-negative CHB patients were prospectively included in a consecutive manner after checking that their liver biopsy results have achieved the agreement of 2 expert histopathologists. CHB was defined as persistent HBsAg positivity for more than 6 months and confirmed by PCR.

### 3.3 Exclusion criteria

- Patients who already started anti-HBV therapy.
- Immunetolerant patients defined as HBeAg positivity with persistent normal ALT in patient <40 years.
- Conditions that might alter liver biochemistry pattern like combined liver diseases, alcohol, and drug intake within 3 months before inclusion.

### 3.4 Laboratory investigations

All laboratory investigations including complete blood count (CBC) and liver biochemistry profile have been done routinely before taking liver biopsy as follow:

- CBC: automated CBC using Sysmex KX-21N (Sysmex Corporation, Kobe, Japan), with assessment of red cell distribution width coefficient of variation (RDWc%).
- Alanine amino transferase (ALT) and aspartate amino transferase (AST): enzymatic rate method.
- Serum bilirubin: Jendrassik and Grof method.
- Albumin: modified Bromocresol green colorimetric method.
- Alkaline phosphatase (ALP): kinetic determination.
- Alpha-fetoprotein (AFP): enzyme-linked immunosorbent assay (ELISA) using immunometric assays (Monobind Incorporation, Lake Forest, CA).

### 3.5 Liver biopsy

Indications for liver biopsy were either elevated ALT > the upper limit of normal (ULN) or HBV viremia ≥ 2000 IU/mL regarding the Egyptian consensus for management of HBeAg-negative CHB adult patients. Selected patients were subjected to ultrasound-guided liver biopsy using an automated 16-gauge Trucut needle within 2 weeks from the initial clinical and laboratory assessment. A minimum length of 2 cm and about 6 portal tracts were checked in each biopsy for accurate staging. All biopsies were stained by hematoxylin and eosin (H&E), and orcein stain. METAVIR score (F0–F4) was used for staging of liver fibrosis by 2 expert histopathologists who were independent, blinded for each other and masked about the biochemical profile of the patients. Patients who had concordant results from the 2 pathologists were included and grouped into: significant fibrosis (F2–F4), advanced fibrosis (F3–F4), and cirrhosis (F4) groups.

### 3.6 Fibrosis indexes

Fibrosis indexes were calculated as listed below and were then compared to the biopsy result.

- S-index: 1000 × GGT ÷ Platelets × Albumin^2^.^[14]^
- RPR: RDWc% ÷ Platelets.^[15]^
- AST/ALT ratio (AAR): AST ÷ ALT.^[16]^
- Fib-4: (Age × AST) ÷ (Platelets × √ALT).^[17]^
- APRI: (AST/ULN ÷ Platelets) × 100, when 40 IU/L was defined as ULN.^[18]^

### 3.7 Statistical analysis

Data were analyzed using analysis of variance (ANOVA), nonparametric test, Chi-squared test, paired *t* test, and pooled *t* test. The overall diagnostic performance for each fibrosis index was evaluated by area under receiver operating characteristics (AUROC) curve analysis. Index with the highest AUROC was validated by calculation of its sensitivity, specificity, positive predictive value (PPV), negative predictive value (NPV) and accuracy for ruling in (confirmation) or ruling out (exclusion) each fibrosis stage. Kappa value (κ) was calculated to measure the significance of agreement between the 2 pathologists. A *P*-value <.05 was considered statistically significant. All statistical analyses were performed using JMP version 13, SAS Institute, Inc., Cary, NC.

## 4 Results

From January 2016 till June 2017, 238 adult naïve HBeAg-negative CHB patients were initially selected, interobservers agreement was achieved in 200 patients who were included in this study (84% agreement, κ = 0.7, *P* < .0001). Based on METAVIR score, patients were classified into 5 fibrosis stages 17%, 34%, 22%, 16%, and 11% were in F0, F1, F2, F3, and F4, respectively. Baseline characteristics of the studied patients are shown in Table 1 which shows statistically significant differences among means of: age (*P* < .008), platelets (*P* = .04), GGT (*P* < .0001), and serum albumin (*P* < .0001) while other variables showed statistically insignificant differences.

**Table 1:**
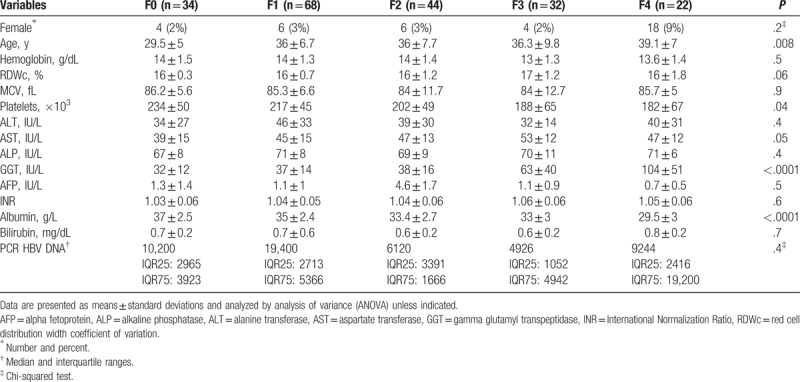
Demographic and laboratory features of studied patients in each stage of fibrosis.

Table 2 shows that means of all studied indexes were statistically significant among F0, F1, F2, F3, and F4 fibrosis stages. Means of S-index were: 0.05, 0.06, 0.06, 0.1, and 0.2 (*P* < .001), RPR: 0.06, 0.06.0.07, 0.08, and 0.08 (*P* < .001), APRI: 0.07, 0.07, 0.6, 0.7, and 0.7 (*P* < .001), AAR: 1.1, 0.8, 0.9, 1.1, and 1 (*P* < .001), and Fib-4: 1.03, 1.04, 1.04, 1.05, and 1.05 (*P* < .001), respectively.

**Table 2:**
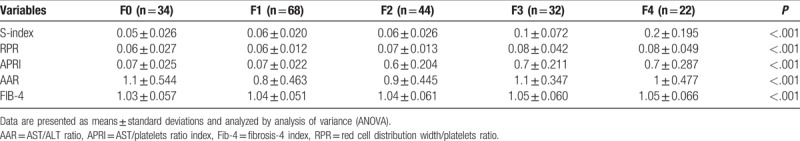
Mean values of the studied indexes in the different fibrosis stages.

As shown in Table 3, S-index could significantly distinguish between F4 versus F0, F4 versus F1, F4 versus F2, F4 versus F3, F3 versus F0, F3 versus F1, and F3 versus F2, both RPR and Fib-4 could significantly distinguish between F4 versus F0, F4 versus F1, F4 versus F2, F3 versus F0, and F3 versus F1, APRI could significantly distinguish between F4 versus F0, F4 versus F1, F3 versus F0, and F3 versus F1, while AAR could only distinguish between F3 versus F1.

**Table 3:**
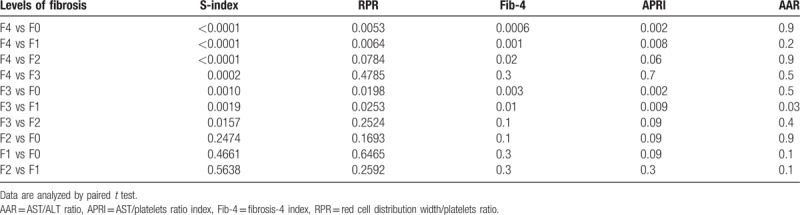
Diagnostic ability of each studied marker in distinguishing between different pairs of fibrosis stages.

Our patients were grouped into 3 fibrosis groups: significant fibrosis (F2–F4, n = 98), advanced fibrosis (F3–F4, n = 54) and cirrhosis (F4, n = 22). Table 4 shows the diagnostic ability of the studied indexes as presented by AUROCs in each group. For significant fibrosis (F2–F4), AUROC of S-index (0.81633) was higher than that of Fib-4 (0.70348), APRI (0.68627), RPR (0.67847), and AAR (0.60084). In advanced fibrosis (F3–F4), AUROC of S-index (0.90538) was better than RPR, Fib-4, APRI, and AAR, and regarding cirrhosis (F4); AUROC of S-index (0.96118) was higher than that RPR (0.62104), Fib-4 (0.61696), APRI (0.57661), and AAR (0.52860).

**Table 4:**
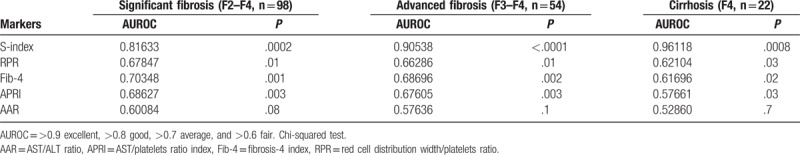
Comparison between AUROCs of the studied indexes for prediction of significant fibrosis (F2–F4), advanced fibrosis (F3–F4), and cirrhosis (F4).

**Figure 1:**
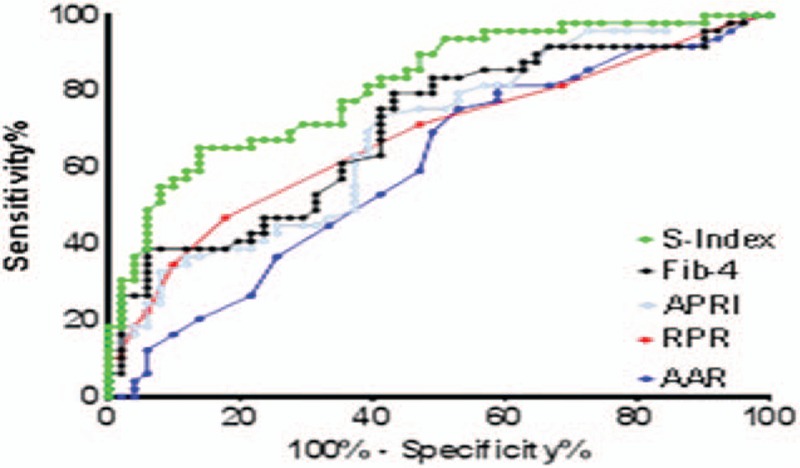
Area under receiver operator characteristics (AUROC) curve of the studied fibrosis indexes in significant fibrosis group (F2–F4, n = 98).

**Figure 2:**
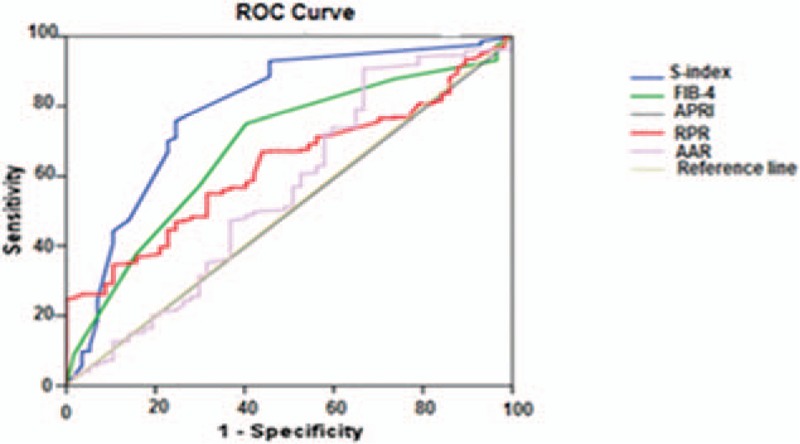
Area under receiver operator characteristics (AUROC) curve of the studied fibrosis indexes in advanced fibrosis group (F3–F4, n = 54).

**Figure 3:**
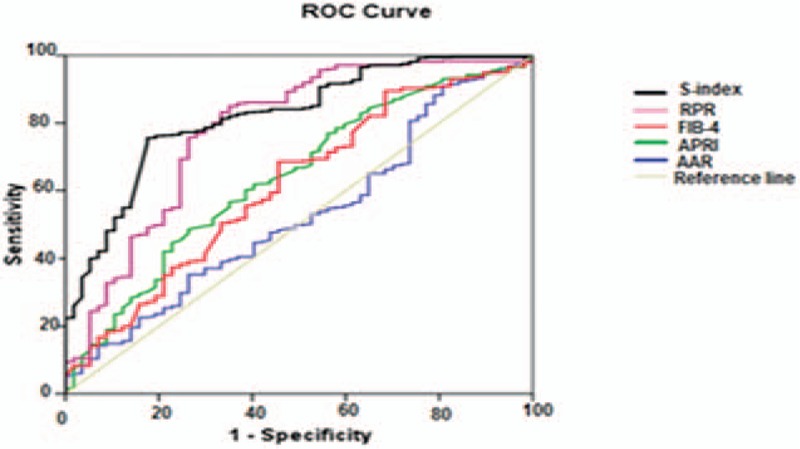
Area under receiver operator characteristics (AUROC) curve of the studied fibrosis indexes in cirrhosis group (F4, n = 22).

As S-index had the highest AUROCs in all fibrosis groups (Figs. 1–3) in our result, we calculated its sensitivity, specificity, PPV, NPV, and accuracy at the best cutoff values for ruling in and ruling out different fibrosis stages (Table 5). In significant fibrosis group, S-index had 94% specificity, 87% PPV, and 68% accuracy for ruling in significant fibrosis at a cutoff value ≥0.3, and 96% sensitivity, 91% NPV, and 67% accuracy for ruling out at a cutoff <1. For advanced fibrosis, the best cutoff for ruling in advanced fibrosis was ≥0.5 with 97% specificity, 80% PPV, and 79% accuracy, and <0.2 for ruling out with 85% sensitivity, 94% NPV, and 83% accuracy. Also, in S-index was highly valuable for diagnosing cirrhosis (F4 group) at a cutoff ≥0.9 with 99% specificity, 75% PPV, and 91% accuracy, and for exclusion of cirrhosis at a cutoff <0.3 with 100% sensitivity, 100% NPV, and 87% accuracy. Accuracy of S-index has increased for confirming higher stages of fibrosis as it was 68% for significant fibrosis, 79% for advanced fibrosis, and 91% for cirrhosis.

**Table 5:**
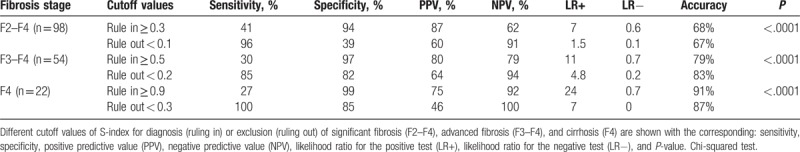
Diagnostic validity of S-index in detection of different fibrosis stages.

**Figure 4:**
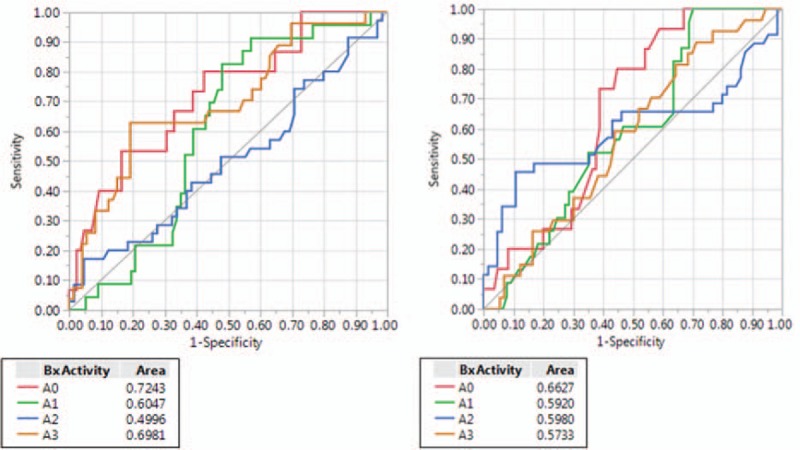
Area under receiver operator characteristics (AUROC) curves of S-index (left side) and AAR (right side) in the different activity groups (A0–A3).

Regarding prediction of inflammatory activity (A0, A1, A2, A3), differences between means of S-index and AAR were statistically significant (Table 6) and both were the only statistically significant predictors with *P* values of .002 and .01, respectively. In A0, A1, A2, and A3 activity groups, AUROCs were 0.7243, 0.6047, 0.4996, 0.6981 for S-index and 0.6627, 0.5920, 0.5980, 0.5733 for AAR, respectively (Fig. 4).

**Table 6:**
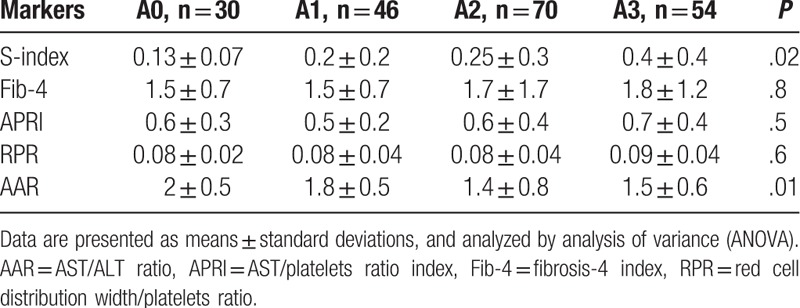
Markers of fibrosis among activity groups (F0–F4).

No complications related to blood sampling or liver biopsy were recorded within 2 hours of close follow up following biopsy taking or within 24 hours after discharge.

## 5 Discussion

The use of noninvasive diagnostic indexes for predicting liver fibrosis and cirrhosis has been validated since 2001 but most studies were conducted in chronic hepatitis C (CHC) patients and only few data are available on their application in CHB patients.^[19–22]^ However, APRI was not an appropriate fibrosis marker in CHB patients as reported in a previous meta-analysis,^[23]^ S-index and RPR were acceptable markers in other studies.^[14,24,25]^ This study aimed to compare validity of 5 noninvasive indexes to liver biopsy in HBeAg-negative CHB patients.

Our result showed that S-index had the highest ability for prediction of all fibrosis stages among all studied indexes, AUROCs of S-index were (0.81633, 0.90538, 0.96118) versus RPR: (0.67847, 0.66286, 0.62104), Fib-4 (0.70348, 0.68696, 0.61696), APRI: (0.68627, 0.67605, 0.57661) and AAR: (0.57636, 0.60084, 0.52860) for significant fibrosis, advanced fibrosis, and cirrhosis, respectively. Also, it was noticed that predictability of S-index steadily increased with higher fibrosis stages as noticed by its higher AUROCs in cirrhosis and advanced fibrosis than in significant fibrosis.

The optimal cutoff for detection of significant fibrosis was ≥0.3 with 94% specificity, 87% PPV, and 68% accuracy which is lower than what has been stated by Zhou et al^[14]^ and Tarigan et al^[26]^ (≥0.5) with 94.8% specificity and 87.8% PPV. Another study,^[27]^ showed that S-index alone had successfully identified significant fibrosis in 87.5% patients with sensitivity 87.5% and specificity 100%, PPV 100%, NPV 66.7%, and AUC 0.93. Also, the cutoff values for exclusion and for detection of advanced fibrosis (F3–F4) were <0.2 with 85% sensitivity, 94% NPV, and 83% accuracy, and ≥0.5 with 97% specificity, 80% PPV, and 79% accuracy, respectively. Regarding cirrhosis the optimal cutoff for its diagnosis was ≥0.9 with 99% specificity, 75% PPV, and 91% accuracy in our result versus ≥1.5 with 98.5% specificity and 80% PPV in Zhou et al.^[14]^ The lower cut-off values in our results may be due to racial differences and restriction of our study to HBeAg-negative CHB patients while in Ref.^[14]^ HBeAg-positive CHB patients were included and in Ref.^[26]^ both CHC and CHB patients were included. Also, our patients were relatively younger with mean age of 35.3 ± 7.7 years versus 48.5 ± 12.70 years compared to Tarigan et al.^[26]^

However mechanisms underlying association between RDW and stage of liver fibrosis in CHB patients are unclear, inflammatory stress, impaired iron mobilization, and iron overload might play key roles in mediating this process as suggested in 2 previous studies.^[27,28]^ Another study among 229 naïve CHB cases reported that RDW and RPR in patients with advanced fibrosis (F3–F4) were significantly higher than that in patients with nonadvanced fibrosis (F0–F2) with *P* values <.05 and <.001, respectively.^[29]^

Our result showed that RPR was a fair predictor for different fibrosis stages as it came first to Fib-4, APRI, and AAR in prediction of cirrhosis with higher AUROC of 0.62104 versus 0.61696, 0.57661, and 0.52860 for Fib-4, APRI, and AAR, respectively. Both Fib-4 and APRI were better than RPR in prediction of significant and advanced fibrosis with AUROCs of 0.70348, 0.68627, and 0.67847 in significant fibrosis and 0.68696, 0.67605, and 0.66286 in advanced fibrosis for Fib-4, APRI, and RPR, respectively. In a study conducted by Chen et al^[15]^ AUROCs of RPR were higher than ours, 0.82 versus 0.68 and 0.88 versus 0.62 for significant fibrosis and cirrhosis, respectively.

This study was limited to fibrosis indexes that depend on routine investigations for CHB patients so important indexes that depend on nonroutine investigations like Forns score and Hepascora were not included. Another important limitation of our study was missing correlation of the surrogate indexes to TE which is a fast, simple, safe, reliable, and widely available procedure,^[30]^ and despite high technical failure rates and certain confounding factors,^[31–33]^ TE is currently a recommended tool for fibrosis assessment in CHB patients with normal or elevated ALT not exceeding 5-fold ULN.^[34]^

In conclusion, S-index has the highest predictability for all fibrosis stages among the studied indexes in HBeAg-negative CHB patients, with higher accuracy in cirrhosis than in the earlier fibrosis stages. However, its validation in a larger number of patients is recommended.
