# Assessment of liver fibrosis in Egyptian chronic hepatitis B patients: A comparative study including 5 noninvasive indexes: Erratum

**DOI:** 10.1097/MD.0000000000010074

**Published:** 2018-03-02

In the article, “Assessment of liver fibrosis in Egyptian chronic hepatitis B patients: A comparative study including 5 noninvasive indexes”,^[1]^ which appeared in Volume 97, Issue 6 of *Medicine*, affiliation b should appear as:

Department of Hepatology, Gastroenterology and Infectious Diseases, Faculty of Medicine, Benha University, Benha, Egypt.
